# Awareness of HIV PrEP, HIV PEP, and Doxy-PEP Among Adults Recruited Through Social Media in Poland: Differences According to Self-Reported Sexual Orientation

**DOI:** 10.3390/healthcare14193177

**Published:** 2026-09-24

**Authors:** Karol Kaleniuk, Natalia Gac, Agata Paduch, Maria Pokorska-Śpiewak, Carlo Bieńkowski

**Affiliations:** 1 Student Scientific Group at the Department of Children’s Infectious Diseases, Medical University of Warsaw, 02-091 Warszawa, Poland; 2 Regional Hospital of Infectious Diseases, Wolska 37, 01-201 Warsaw, Poland; 3 Department of Children’s Infectious Diseases, Medical University of Warsaw, Wolska 37, 02-091 Warsaw, Poland; 4 Department of Adults’ Infectious Diseases, Medical University of Warsaw, Wolska 37, 02-091 Warsaw, Poland

**Keywords:** HIV PrEP, HIV PEP, Doxy-PEP, sexual minorities, STI prevention

## Abstract

**Introduction:** HIV pre-exposure prophylaxis (HIV PrEP) and HIV post-exposure prophylaxis (HIV PEP) are established preventive strategies against HIV, while doxycycline post-exposure prophylaxis (doxy-PEP) is used to prevent bacterial sexually transmitted infections (STIs). Awareness, access, and use of these methods may differ between heterosexual persons and sexual minority individuals (SMIs). This study aimed to assess awareness of HIV PrEP, HIV PEP, and doxy-PEP among adults in Poland recruited through social media and to compare awareness according to self-reported sexual orientation. **Materials and methods:** We conducted a cross-sectional survey of Polish adults recruited through social media platforms. Collected data included demographic characteristics, sexual behavior, awareness and knowledge of HIV PrEP, HIV PEP, and doxy-PEP, willingness to use preventive measures, and access-related knowledge. Comparative analyses were performed between heterosexual persons and SMIs. **Results:** We collected 204 questionnaires. Of these, 196 were included in the final analysis, while 8 (3.9%) respondents with missing or unspecified sexual orientation were excluded. The majority of participants were heterosexual 59.7%, with 31.1% identifying as homosexual and 9.2% as bisexual. Of the 196 participants, 54.6% were male, and 45.4% were female. The median respondent age was 29.5 years (IQR: 25.0–36.8 years), and most respondents had higher education (61.7%). Compared to heterosexual respondents, SMIs reported more sexual partners (median 4 vs. 1; *p* < 0.001), more often reported condomless sexual intercourse or condom failure with a partner of unknown serological status (69.6% vs. 26.5%; *p* < 0.001), and more often had been tested for HIV (74.7% vs. 31.6%; *p* < 0.001). Moreover, SMIs were more likely to report STIs, particularly syphilis (*p* = 0.004), chlamydia (*p* = 0.004), gonorrhea (*p* < 0.001), and pubic lice (*p* = 0.028). SMIs were more aware of HIV prevention strategies, including being more familiar with HIV PEP (77.2% vs. 30.8%; *p* < 0.001) and doxy-PEP (54.4% vs. 18%; *p* < 0.001). In univariable analyses, SMI participants had higher prevalence of doxy-PEP awareness (PR 3.03, 95% CI: 1.96–4.69; *p* < 0.001) and self-reported STI history (PR 9.43, 95% CI: 3.90–22.83; *p* < 0.001). In multivariable analyses, previous HIV testing was independently associated with awareness of HIV PrEP (aPR 1.97, 95% CI: 1.32–2.93; *p* < 0.001), HIV PEP (aPR 1.89, 95% CI: 1.20–2.96; *p* = 0.006), and doxy-PEP (aPR 2.12, 95% CI: 1.10–4.07; *p* = 0.025), while SMIs remained independently associated with HIV PEP awareness (aPR 1.60, 95% CI: 1.04–2.45; *p* = 0.031). **Conclusions:** Despite greater awareness of HIV and prevention of bacterial STIs, SMIs reported more frequent sexual encounters associated with potential exposure to STIs and a higher self-reported history of STI diagnoses. Previous HIV testing was consistently associated with greater awareness of HIV PrEP, HIV PEP, and doxy-PEP, highlighting HIV testing encounters as an important opportunity for integrated prevention education. These findings highlight the need for further research on the clinical eligibility, acceptability, and implementation of doxy-PEP among individuals meeting guideline-defined eligibility criteria in Poland.

## 1. Introduction

Sexually transmitted infections (STIs) remain a major global public health issue and continue to pose a significant epidemiological burden in Poland [1,2]. New cases of STIs continue to be reported annually. In 2024, a total of 2257 new human immunodeficiency virus (HIV) infections were diagnosed in Poland, corresponding to an incidence of 6.01 per 100,000 population, with men accounting for 76.5% of all newly diagnosed cases [3]. Sexual contact between men remained the most frequently reported route of HIV transmission [3]. The same year, 3258 new syphilis cases were reported, corresponding to an incidence of 8.67 per 100,000 population, as well as 1151 cases of gonorrhea (3.06 per 100,000) and 1186 cases of chlamydia (3.16 per 100,000), confirming that both viral and bacterial STIs continue to represent a substantial epidemiological burden in Poland [3].

Several pharmacological preventive strategies are currently available to reduce the risk of acquiring HIV and bacterial STIs. HIV pre-exposure prophylaxis (HIV PrEP) involves the use of antiretroviral drugs by HIV-negative individuals to reduce the risk of infection before exposure. The standard oral regimen consists of a fixed-dose combination of tenofovir disoproxil fumarate and emtricitabine [4]. In Poland, only oral formulations are currently available, whereas several European countries have already introduced long-acting injectable PrEP options [5]. Injectable cabotegravir is administered every two months, whereas lenacapavir, recently evaluated as a long-acting preventive option, is administered every six months [6,7].

HIV post-exposure prophylaxis (HIV PEP) consists of short-term antiretroviral treatment initiated after potential exposure to HIV, including sexual, occupational, and non-occupational exposures. HIV PEP should be initiated as soon as possible, alater than 72 h after the exposure, and continued for 28 days using a recommended three-drug antiretroviral regimen [4].

In recent years, doxycycline post-exposure prophylaxis (doxy-PEP) has gained increasing interest as a preventive strategy against bacterial STIs [8]. In 2025, new European AIDS Clinical Society recommendations included doxycycline post-exposure prophylaxis for selected individuals at increased risk of bacterial STIs [9]. This approach consists of a single 200 mg oral dose of doxycycline, taken within 24–72 h after sexual exposure [4]. This intervention was efficacious in reducing the incidence of bacterial STIs, including syphilis, chlamydia, and gonorrhea; however, it has only been proven to be effective in men who have sex with men (MSM) and transsexual women. An 83% combined reduction in syphilis and chlamydia infections has been reported [10]. Although Polish guidelines include doxy-PEP as a preventive strategy, doxycycline is not currently approved for this indication in Poland and its use remains off-label [4,11].

Despite the increasing availability of STI preventive methods, awareness and access to reliable information remain uneven across populations [12]. Knowledge of where and when preventive interventions should be implemented may influence their uptake [13]. However, in Poland, data on awareness of HIV PrEP, HIV PEP, and particularly doxy-PEP across different sexual orientation groups remain limited.

This study aimed to assess awareness of HIV PrEP, HIV PEP, and doxy-PEP among adults in Poland recruited through social media and to compare awareness according to self-reported sexual orientation.

## 2. Materials and Methods

### 2.1. Study Design and Participants

The questionnaire was created in Polish using Google Forms (Google LLC, Mountain View, CA, USA) and subsequently distributed via social media platforms, including Facebook groups focused on lifestyle, sexual health, No Taboo discussions, and LGBTQ+ communities, as well as Instagram. The same recruitment message was used across all platforms. The survey invitation was posted once in each selected social media group or platform during the recruitment period. Participation was voluntary, and no paid advertisements or sponsored posts were used to promote the survey. Before distribution, the questionnaire was reviewed by the study authors and several individuals from the medical community to assess its clarity and comprehensibility. Minor revisions were made based on their feedback before the final version was distributed.

The questionnaire used conditional branching. Questions applicable to a participant based on previous responses were mandatory, whereas non-applicable questions were automatically skipped. Although participant authentication was not required, the completed questionnaires were reviewed for potential duplicate entries, and no apparent duplicate responses were identified.

Sexual minority individuals (SMIs) were defined as participants who reported a homosexual or bisexual sexual orientation.

### 2.2. Collected Data

Data were collected between 1 December, 2025, and 31 January, 2026, and included sociodemographic characteristics (sex, age, place of residence, education, and sexual orientation), sexual activity, number of sexual partners, HIV testing history, previous sexually transmitted infections, and knowledge of HIV transmission, as well as awareness of and attitudes toward preventive methods, including condom use, HIV PrEP, HIV PEP, and doxy-PEP.

For this study, a risky sexual encounter was defined as condomless sexual intercourse or condom failure during sexual intercourse with a partner of unknown HIV serostatus, including encounters occurring under the influence of psychoactive substances.

### 2.3. Inclusion Criteria

Adult respondents (≥18 years) residing in Poland were eligible to participate, and completion of the questionnaire following presentation of the study information was considered as informed consent to participate.

### 2.4. Exclusion Criteria

Respondents younger than 18 years and questionnaires with missing or unspecified information on sexual orientation were excluded from the final analysis.

### 2.5. Statistical Analysis

No formal sample size calculation was performed because the study was exploratory in nature. Participant recruitment continued throughout the predefined study period. Continuous variables were summarized as medians with interquartile ranges (IQR) and compared using the Mann–Whitney U test. Categorical variables were presented as frequencies and percentages and compared using the chi-square test or Fisher’s exact test, as appropriate.

To identify factors independently associated with the analyzed binary outcomes (sexual minority status, previous HIV testing, history of sexually transmitted infections, awareness of HIV PrEP, HIV PEP, and doxy-PEP), univariable and multivariable Poisson regression models with a log link and robust (sandwich) variance estimator were applied. Because the outcomes analyzed were relatively common, this approach was chosen to directly estimate prevalence ratios (PRs) and adjusted prevalence ratios (aPRs) with corresponding 95% confidence intervals (95% CIs), rather than odds ratios obtained from logistic regression. Variables included in the multivariable models were selected a priori based on clinical relevance and previous literature and comprised age, sex, number of sexual partners, educational level, previous HIV testing, and history of sexually transmitted infections, as appropriate for each outcome.

All statistical tests were two-sided, and a *p*-value < 0.05 was considered statistically significant. Statistical analyses were performed using R software, version 4.6.1 (R Foundation for Statistical Computing, Vienna, Austria).

### 2.6. Ethical Statement

The study was conducted in accordance with the Declaration of Helsinki and acknowledged by the Medical University of Warsaw’s Bioethics Committee. Document number: AKBE/93/2026. Before starting the survey, each participant received information about the purpose of the study, anonymity, and the right to withdraw at any stage without providing a reason. Completion of the questionnaire was considered to imply informed consent to participate in the study. Participants did not receive any financial compensation or other material benefits for taking part in the study.

## 3. Results

### 3.1. Participant Characteristics and Sexual Health History

A total of 204 questionnaires were collected. Eight responses (3.9%) were excluded due to missing or unspecified sexual orientation, and 196 questionnaires were included in the final analysis. Among the analyzed participants, 59.7% identified themselves as heterosexual, 31.1% as homosexual, and 9.2% as bisexual. Overall, 54.6% of respondents were male, and 45.4% were female. The median age was 29.5 years (IQR: 25.0–36.8), and 61.7% of participants had higher education. SMIs were more likely to live in cities with more than 250,000 inhabitants than heterosexual respondents (77.2% vs. 46.2%, *p* < 0.001).

SMIs reported significantly higher sexual activity compared to heterosexual respondents. The median number of sexual partners in the past 12 months was 4 (IQR: 1–10) in the SMI group and 1 (IQR: 1–1) among heterosexual individuals (*p* < 0.001). Sexual encounters with increased risk of STI exposure were reported more frequently by SMIs than heterosexual respondents (69.6% vs. 26.5%, *p* < 0.001). Consultations due to the aforementioned sexual encounters were more frequently reported by SMIs than heterosexual respondents (46.8% vs. 6.8%, *p* < 0.001). A history of at least one STI was reported more often among SMIs than heterosexual respondents (49.3% vs. 5.0%, *p* < 0.0001). Specific infections were also more frequently reported by SMIs: syphilis (8.45% vs. 0%, *p* = 0.004), chlamydia (11.3% vs. 1%, *p* = 0.004), gonorrhea (12.7% vs. 0%, *p* < 0.0001), and pubic lice (5.6% vs. 0%, *p* = 0.028). HIV testing had been performed more frequently among SMIs than heterosexual respondents (74.7% vs. 31.6%, *p* < 0.0001). Similarly, HIV PEP use was reported more frequently by SMIs (26.6% vs. 0.85%, *p* < 0.0001) (Table 1).

### 3.2. Awareness and Knowledge of HIV and STI Prevention Strategies

Knowledge regarding possible routes of HIV transmission was similar between both groups. However, SMIs more frequently identified oral intercourse as a potential mode of HIV transmission (87.3% vs. 70.1%, *p* = 0.005). Greater familiarity with prevention strategies was more often observed among SMIs than heterosexual respondents. SMIs were more likely to know about HIV prevention methods (87.3% vs. 46.2%, *p* < 0.001). Awareness of HIV PrEP was also more frequently reported (83.5% vs. 37.6%, *p* < 0.001), as was awareness of HIV PEP (77.2% vs. 30.8%, *p* < 0.001) and doxy-PEP (54.4% vs. 18.0%, *p* < 0.001) by SMIs. Knowledge of where to access HIV PrEP, PEP, or doxy-PEP was more frequently reported by SMIs (55.7% vs. 33.3%, *p* = 0.002), and higher self-rated sexual health knowledge was observed in SMIs (median 8 (IQR: 6–8) vs. 6 (IQR: 5–8), *p* = 0.002).

### 3.3. Univariable Analysis

In univariable Poisson regression analyses, belonging to the SMI group was significantly associated with all study outcomes. The strongest association was observed for a self-reported history of STI, which was 9.43-fold more prevalent among SMI participants than among heterosexual participants (PR 9.43, 95% CI: 3.90–22.83; *p* < 0.001). SMI participants also had a higher prevalence of previous HIV testing (PR 2.27, 95% CI: 1.72–2.99; *p* < 0.001) and greater awareness of HIV PrEP, HIV PEP, and doxy-PEP, with the strongest association observed for doxy-PEP awareness (PR 3.03, 95% CI: 1.96–4.69; *p* < 0.001). These findings indicate a consistent pattern of greater engagement with HIV testing and greater awareness of HIV and STI prevention strategies among SMI participants in the univariable analyses (Table 2).

### 3.4. Multivariable Analysis

In multivariable Poisson regression analysis, previous HIV testing was independently associated with greater awareness of HIV PrEP, HIV PEP, and doxy-PEP, with previously tested participants having a 1.97-fold, 1.89-fold, and 2.12-fold higher prevalence of awareness, respectively (aPR 1.97, 95% CI: 1.32–2.93; *p* < 0.001; aPR 1.89, 95% CI: 1.20–2.96; *p* = 0.006; and aPR 2.12, 95% CI: 1.10–4.07; *p* = 0.025). For HIV PEP awareness, SMI was also independently associated with a 1.60-fold higher prevalence of awareness (aPR 1.60, 95% CI: 1.04–2.45; *p* = 0.031), as was a higher number of sexual partners (aPR 1.00, 95% CI: 1.00–1.01; *p* = 0.021). A higher number of sexual partners was also independently associated with a 1.01-fold higher prevalence of doxy-PEP awareness (aPR 1.01, 95% CI: 1.00–1.02; *p* < 0.001). Regarding a history of STI, SMI was associated with a 3.68-fold higher prevalence of reporting a history of STI (aPR 3.68, 95% CI: 1.14–11.83; *p* = 0.029), while a higher number of sexual partners was associated with a 1.01-fold higher prevalence (aPR 1.01, 95% CI: 1.00–1.02; *p* = 0.027). Previous HIV testing showed the strongest association with a history of STI, being associated with a 13.68-fold higher prevalence of reporting a history of STI (aPR 13.68, 95% CI: 2.17–86.36; *p* = 0.005) (Table 3).

## 4. Discussion

SMIs represent a population disproportionately affected by HIV and bacterial STIs [14]. Previous studies have shown that this group more frequently reports higher numbers of sexual partners, more frequent sexual encounters with increased risk of STI acquisition, and a greater prevalence of previous STI diagnoses [15]. Therefore, sexual minorities are increasingly recognized as a priority population for targeted preventive interventions, including improved access to HIV PrEP, HIV PEP, and doxy-PEP, as reflected in international public health guidelines [16].

The study demonstrated disparities in HIV and bacterial STI prevention strategy awareness between SMIs and heterosexual individuals (Table 1). Previous Polish studies conducted among university students demonstrated insufficient knowledge regarding HIV and STI prevention, including limited awareness of HIV PrEP and HIV PEP, low testing rates despite sexual encounters associated with potential STI exposure, and poor familiarity with preventive campaigns [17,18]. However, these studies did not differentiate participants according to their sexual orientation and were limited to student populations. In our study, we differentiated the participants by declared sexual orientation. SMIs showed greater awareness of HIV and prevention strategies of bacterial STIs compared to heterosexual individuals. This finding may reflect greater exposure to sexual health-related information and HIV prevention services among SMI participants. However, the multivariable analyses suggest that some of these differences may be explained by other characteristics included in the models. After adjustment for confounders, SMI participants were 1.60-fold more likely to report HIV PEP awareness (aPR 1.60, 95% CI: 1.04–2.45; *p* = 0.031), whereas the associations between SMI and PrEP or doxy-PEP awareness were no longer statistically significant.

STIs may lead to complications extending beyond acute disease, including long-term health impairment, reproductive disorders, and adverse pregnancy outcomes [8,19].

In addition, delayed diagnosis of HIV remains a major public health concern. Across the WHO European Region, more than half (52% for the total WHO European Region, 53% for EU/EEA) of HIV diagnoses are made late, leading to higher morbidity and an increased risk of AIDS-related death [20]. In our study, heterosexual individuals demonstrated lower awareness of HIV and prevention strategies of bacterial STIs, reported lower rates of STI testing, and had fewer sexual partners within the previous year compared to SMI participants. In the univariable analysis, belonging to the SMI group was also associated with previous HIV testing (PR 2.27, 95% CI: 1.72–2.99; *p* < 0.001). In the multivariable analysis, SMI participants had a 1.44-fold higher prevalence of previous HIV testing, although this association was not statistically significant (aPR 1.44, 95% CI: 0.99–2.10; *p* = 0.058), while a history of STI was independently associated with previous HIV testing (aPR 1.57, 95% CI: 1.25–1.97; *p* < 0.001). One possible explanation for lower engagement in HIV testing among some participants may be a lower perceived risk of STI acquisition, which could reduce engagement in preventive behaviors.

At the same time, SMIs in our study reported a greater number of sexual partners and more frequent engagement in sexual encounters associated with potential exposure to STIs. In addition, a history of syphilis, chlamydia, gonorrhea, and pubic lice infestation was more often reported by SMIs (respectively: 8.45%, *p* = 0.004; 11.27%, *p* = 0.004; 12.68%, *p* < 0.001; and 5.63%, *p* = 0.028). Previous studies have shown that greater familiarity with STIs and preventive methods does not necessarily translate into safer sexual practices [21]. Similarly, in our study, SMIs demonstrated higher awareness while still engaging in higher-risk behaviors. Importantly, SMI participants had a 3.68-fold higher prevalence of a history of STI after adjustment for confounders (aPR 3.68, 95% CI: 1.14–11.83; *p* = 0.029). A greater number of sexual partners was also associated with a higher prevalence of STI history (aPR 1.01, 95% CI: 1.00–1.02; *p* = 0.027), while previous HIV testing showed the strongest association, being associated with a 13.68-fold higher prevalence of a reported STI history (aPR 13.68, 95% CI: 2.17–86.36; *p* = 0.005). This finding should be interpreted cautiously, as differences in testing frequency may have influenced STI detection. Individuals who undergo HIV and STI testing more frequently may have more opportunities for STI diagnosis and reporting. However, it cannot be clearly determined whether greater awareness preceded STI acquisition or developed because of previous STI-related experiences.

Greater familiarity with HIV PrEP among SMIs may be associated with greater exposure to sexual health-related information and HIV prevention services. Other factors potentially contributing to greater awareness may include previous HIV testing, greater numbers of sexual partners, and more frequent contact with sexual health services, which were also associated with awareness in our multivariable analyses. Although SMIs demonstrated greater awareness of HIV PrEP in the univariable analysis, this association was no longer statistically significant after multivariable adjustment (aPR 1.46, 95% CI: 1.00–2.12; *p* = 0.050). In contrast, participants with previous HIV testing were 1.97-fold more likely to report HIV PrEP awareness (aPR 1.97, 95% CI: 1.32–2.93; *p* < 0.001). Therefore, the higher PrEP awareness observed among SMIs in the univariable analysis may be influenced by differences in other characteristics included in the multivariable model. The differences observed in specific questions regarding the limitations of PrEP should also be interpreted cautiously, as the reported differences did not reach statistical significance. Previous studies have shown that practical knowledge regarding HIV PrEP efficacy, adherence, and limitations may play an important role in willingness to use and effectively implement HIV PrEP-based prevention strategies [13].

In our study, SMI participants showed higher awareness and better understanding of when HIV PEP should be used. Since HIV PEP is intended for use after potential HIV exposure, this may reflect more frequent encounters with situations involving potential HIV exposure and greater familiarity with post-exposure prevention. Previous studies among men who have sex with men have linked higher HIV PEP awareness with prior HIV testing, greater HIV-related knowledge, and increased engagement with HIV prevention efforts [22]. Consistent with these findings, SMI participants were 1.60-fold more likely to report HIV PEP awareness after adjustment for confounders (aPR 1.60, 95% CI: 1.04–2.45; *p* = 0.031). Previous HIV testing was also associated with greater HIV PEP awareness (aPR 1.89, 95% CI: 1.20–2.96; *p* = 0.006), while a greater number of sexual partners showed a smaller but statistically significant association (aPR 1.00, 95% CI: 1.00–1.01; *p* = 0.021).

Our study also demonstrated higher familiarity with doxy-PEP among SMI participants (Table 1). Doxy-PEP was recently included in the 2025 EACS guidelines for selected high-risk populations, reflecting a growing interest in biomedical prevention strategies of STIs [9]. However, overall awareness of doxy-PEP in our study remained low, suggesting that this approach has not yet reached broader populations. This may partly reflect its relatively recent introduction, as well as ongoing concerns regarding antimicrobial resistance. Repeated use of doxycycline for post-exposure prophylaxis may contribute to the development of resistant bacterial STI strains, including *Neisseria gonorrhoeae*, potentially limiting the long-term effectiveness of this strategy [23]. Although SMIs showed greater doxy-PEP awareness in the univariable analysis, this association was no longer statistically significant after adjustment for confounders (aPR 1.44, 95% CI: 0.82–2.50; *p* = 0.201). In contrast, a greater number of sexual partners was associated with greater doxy-PEP awareness (aPR 1.01, 95% CI: 1.00–1.02; *p* < 0.001), while previous HIV testing was associated with a more than two-fold higher prevalence of doxy-PEP awareness (aPR 2.12, 95% CI: 1.10–4.07; *p* = 0.025).

Effective prevention of HIV and other STIs depends not only on awareness of available prophylactic methods but also on equitable access to preventive services and appropriate sexual health education. Previous studies have emphasized that the benefits of HIV PrEP cannot be fully realized without ensuring equitable access to prevention services across different populations [24]. In addition, comprehensive sexual health education has been shown to improve related knowledge, increase engagement in preventive behaviors, and support informed use of biomedical prevention strategies [25]. Together, these findings highlight the importance of combining accessible prevention services with effective sexual health education to reduce incidence of HIV and STIs.

Taken together, our findings highlight differences in awareness and knowledge between SMIs and heterosexual individuals and underscore the need for targeted and population-specific health education strategies. Although belonging to the SMI group was associated with greater awareness of several HIV and STI prevention strategies in the univariable analyses, the multivariable results indicate that previous HIV testing was a consistent independent factor associated with awareness of PrEP, PEP, and doxy-PEP, while SMIs remained independently associated only with PEP awareness among the three preventive strategies examined. Improving access to information about HIV PrEP and HIV PEP in the general population should be a priority. Given the low awareness of doxy-PEP identified in this study, further research is warranted to evaluate the potential role of doxy-PEP in Poland, particularly among populations at increased risk of bacterial sexually transmitted infections. The findings may also help inform future clinical guidance and public health communication.

## 5. Limitations

This study has several limitations that should be considered when interpreting the findings. First, the use of an online survey and convenience sampling through social media platforms may have introduced selection bias, as participation was limited to individuals with internet access and adequate digital literacy. This recruitment strategy may have preferentially recruited younger, more health-conscious, and more socially engaged individuals. Consequently, the study sample is not representative of the general adult population of Poland or of all sexual minority individuals in Poland, and the findings should therefore be interpreted with caution. Second, the cross-sectional design precludes conclusions about causality between the variables examined. Additionally, all data were self-reported, which introduces the possibility of recall bias and social desirability bias, particularly in relation to sensitive topics including sexual behavior and history of STIs. Although no apparent duplicate responses were identified during data review, the anonymous nature of the survey meant that duplicate responses could not be formally excluded. The overrepresentation of urban residents and men among SMIs may have further limited the external validity and generalizability of the findings. Although multivariable analyses were performed to account for several potential confounding factors, residual confounding by unmeasured variables cannot be excluded.

Despite these limitations, the study has notable strengths, including the simultaneous assessment of awareness of three preventive strategies (HIV PrEP, HIV PEP, and doxy-PEP), as well as a direct comparison between heterosexual individuals and SMIs in the Polish context, where data remain limited. Further research involving larger and more representative populations is needed to confirm and extend these findings.

## 6. Conclusions

SMIs demonstrated greater knowledge of HIV prevention strategies, including HIV PrEP, HIV PEP, and prevention of bacterial STIs with doxy-PEP. However, they were also more likely to engage in sexual encounters associated with potential STI exposure and to report sexually transmitted infections. These findings show that greater awareness may coexist with reported sexual risk behaviours; however, the cross-sectional design does not allow us to establish a temporal or causal relationship between awareness and sexual behaviour. Previous HIV testing emerged as the most consistent factor associated with greater awareness of HIV PrEP, HIV PEP, and doxy-PEP. Broader sexual health education strategies, alongside targeted interventions for high-risk populations, are needed to improve prevention outcomes. Future studies should assess awareness, clinical eligibility, acceptability, and implementation requirements for doxy-PEP among individuals at increased risk of bacterial STIs in Poland.

## Figures and Tables

**Table 1:** Sociodemographic characteristics, sexual behaviors, and HIV/STI-related knowledge, including HIV PrEP, HIV PEP, and Doxy-PEP, among Polish adults stratified by self-reported sexual orientation.

Variable	All Participants	Heterosexual Individuals	SMIs	*p*-Value
**N**	**196**	**117**	**79**	**-**
**Part I: Sociodemographic data**				
**Sex [n/N (%)]**				
male	107/196 (54.59%)	40/117 (34.19%)	67/79 (84.81%)	<0.001
female	89/196 (45.41%)	77/117 (65.81%)	12/79 (15.19%)	
**Age**				
median [IQR]	29.5 [25–36.75]	27 [24–38.5]	31 [27–36]	0.132
**Place of residence [n/N (%)]**				
village	31/196 (15.82%)	26/117 (22.22%)	5/79 (6.33%)	<0.001
city < 50k	14/196 (7.14%)	8/117 (6.84%)	6/79 (7.59%)	
city 50–250k	36/196 (18.37%)	29/117 (24.79%)	7/79 (8.86%)	
city > 250k	115/196 (58.67%)	54/117 (46.15%)	61/79 (77.22%)	
**Education [n/N (%)]**				
elementary	2/196 (1.02%)	2/117 (1.71%)	0/79	0.137
secondary	20/196 (10.20%)	14/117 (11.97%)	6/79 (7.59%)	
university student	53/196 (27.04%)	36/117 (30.77%)	17/79 (21.52%)	
higher	121/196 (61.74%)	65/117 (55.56%)	56/79 (70.89%)	
**Part II: Sexual practices**				
**Do you know what a risky sexual encounter is? [n/N (%)]**				
yes	194/196 (98.98%)	115/117 (98.29%)	79/79 (100%)	0.243
no	2/196 (1.02%)	2/117 (1.71%)	0/79	
**Are you sexually active? [n/N (%)]**				
yes	172/196 (87.76%)	101/117 (86.32%)	71/79 (89.87%)	0.457
no	24/196 (12.24%)	16/117 (13.68%)	8/79 (10.13%)	
**How many sexual partners have you had in the last 12 months?**				
median [IQR]	1 [1–3]	1 [1–1]	4 [1–10]	<0.001
**Have you ever been tested for HIV? [n/N (%)]**				
yes	96/196 (48.98%)	37/117 (31.62%)	59/79 (74.68%)	<0.001
no	100/196 (51.02%)	80/117 (68.38%)	20/79 (25.32%)	
**Have you ever had an STI? [n/N (%)]**				
yes	40/172 (23.26%)	5/101 (4.95%)	35/71 (49.30%)	<0.001
no	122/172 (70.93%)	88/101 (87.13%)	34/71 (47.88%)	
I don’t know	10/172 (5.81%)	8/101 (7.92%)	2/71 (2.82%)	
**Have you had an STI in the last 12 months? [n/N (%)]**				
yes	15/172 (8.72%)	2/101 (1.98%)	13/71 (18.31%)	<0.001
no	149/172 (86.63%)	93/101 (92.08%)	56/71 (78.87%)	
I don’t know	8/172 (4.65%)	6/101 (5.94%)	2/71 (2.82%)	
**What STIs have you ever had? [n/N (%)]**				
HIV	2/172 (1.16%)	0/101	2/71 (2.82%)	0.085
Hepatitis B	2/172 (1.16%)	1/101 (0.99%)	1/71 (1.41%)	1
Hepatitis C	2/172 (1.16%)	0/101	2/71 (2.82%)	0.085
Syphilis	6/172 (3.48%)	0/101	6/71 (8.45%)	0.004
N. gonorrhoeae	9/172 (5.22%)	0/101	9/71 (12.68%)	<0.001
C. trachomatis	9/172 (5.22%)	1/101 (0.99%)	8/71 (11.27%)	0.004
HSV-1or 2	4/172 (2.32%)	2/101 (1.98%)	2/71 (2.82%)	1
Trichomonas vaginalis	0/172	0/101	0/71	1
Pubic lice	4/172 (2.32%)	0/101	4/71 (5.63%)	0.028
Molluscum contagiosum	0/172	0/101	0/71	1
Mpox	0/172	0/101	0/71	1
M. genitalium	0/172	0/101	0/71	1
HPV	3/172 (1.74%)	1/101 (0.99%)	2/71 (2.82%)	0.570
**Have you ever had a sexual encounter previously defined as risky? [n/N (%)]**				
yes	86/196 (43.88%)	31/117 (26.50%)	55/79 (69.62%)	<0.001
no	110/196 (56.12%)	86/117 (73.50%)	24/79 (30.38%)	
**Have you ever been consulted by a physician due to a sexual encounter previously defined as risky? [n/N (%)]**				
yes	45/196 (22.96%)	8/117 (6.84%)	37/79 (46.84%)	<0.001
no	151/196 (77.04%)	109/117 (93.16%)	42/79 (53.16%)	
**Have you ever used HIV PEP? [n/N (%)]**				
yes	22/196 (11.22%)	1/117 (0.85%)	21/79 (26.58%)	<0.001
no	174/196 (88.78%)	116/117 (99.15%)	58/79 (73.42%)	
**Do you know HIV transmission routes? [n/N (%)]**				
yes	195/196 (99.49%)	117/117 (100%)	78/79 (98.73%)	0.403
no	1/196 (0.51%)	0/117	1/79 (1.27%)	
**How can you acquire HIV infection? [n/N (%)]**				
anal intercourse	179/196 (91.33%)	103/117 (88.03%)	76/79 (92.20%)	0.068
vaginal intercourse	185/196 (94.39%)	111/117 (94.87%)	74/79 (93.67%)	0.759
oral–genital contact	151/196 (80.10%)	82/117 (70.09%)	69/79 (87.34%)	0.005
kiss	21/196 (10.72%)	13/117 (11.11%)	8/79 (10.13%)	1
touching	6/196 (3.06%)	4/117 (3.42%)	2/79 (2.53%)	1
toilet seat	11/196 (5.61%)	9/117 (7.69%)	2/79 (2.53%)	0.205
damaged skin	175/196 (89.29%)	105/117 (89.74%)	70/79 (88.61%)	0.817
droplets	13/196 (6.63%)	10/117 (8.55%)	3/79 (3.80%)	0.248
needles	182 (92.86%)	108/117 (92.31%)	74/79 (93.67%)	0.784
**Do condoms protect against HIV acquisition? [n/N (%)]**				
yes	171/196 (87.24%)	100/117 (85.47%)	71/79 (89.87%)	0.185
no	16/196 (8.16%)	9/117 (7.69%)	7/79 (8.86%)	
I don’t know	9/196 (4.60%)	8/117 (6.84%)	1/79 (1.27%)	
**Part III: Pharmacological prevention methods**				
**Are there pharmacological methods to prevent HIV infection? [n/N (%)]**				
yes	123/196 (62.76%)	54/117 (46.15%)	69/79 (87.34%)	<0.001
no	23/196 (11.73%)	19/117 (16.24%)	4/79 (5.06%)	
I don’t know	50/196 (25.51%)	44/117 (37.61%)	6/79 (7.59%)	
**Have you ever heard of HIV PrEP? [n/N (%)]**				
yes	110/196 (56.12%)	44/117 (37.61%)	66/79 (83.54%)	<0.001
no	86/196 (43.88%)	73/117 (62.39%)	13/79 (16.46%)	
**HIV PrEP is recommended for seronegative individuals at increased risk of HIV infection.** **[n/N (%)]**				
yes	163/196 (83.16%)	91/117 (77.78%)	72/79 (91.14%)	0.036
no	3/196 (1.53%)	3/117 (2.56%)	0/79	
I don’t know	30/196 (15.31%)	23/117 (19.66%)	7/79 (8.86%)	
**Is HIV PrEP intended for use only among men? [n/N (%)]**				
yes	2/196 (1.02%)	0/117	2/79 (2.53%)	0.079
no	140/196 (71.43%)	80/117 (68.38%)	60/79 (75.95%)	
I don’t know	54/196 (27.55%)	37/117 (31.62%)	17/79 (21.52%)	
**Does HIV PrEP protect against infections other than HIV? [n/N (%)]**				
yes	11/196 (5.61%)	9/117 (7.69%)	2/79 (2.53%)	<0.001
no	116/196 (59.18%)	56/117 (47.86%)	60/79 (75.95%)	
I don’t know	69/196 (35.21%)	52/117 (44.44%)	17/79 (21.52%)	
**Should condoms still be used when taking HIV PrEP? [n/N (%)]**				
yes	17/196 (8.67%)	10/117 (8.55%)	7/79 (8.86%)	0.057
no	156/196 (79.59%)	88/117 (75.21%)	68/79 (86.08%)	
I don’t know	23/196 (11.74%)	19/117 (16.24%)	4/79 (5.06%)	
**Does HIV PrEP prevent pregnancy? [n/N (%)]**				
yes	1/196 (0.51%)	0/117	1/79 (1.27%)	0.168
no	175/196 (89.29%)	102/117 (87.18%)	73/79 (92.41%)	
I don’t know	20/196 (10.20%)	15/117 (12.82%)	5/79 (6.33%)	
**Which form of HIV PrEP would you prefer if you were to use it? [n/N (%)]**				
daily	38/196 (19.39%)	27/117 (23.08%)	11/79 (13.92%)	0.424
on demand	56/196 (28.57%)	31/117 (26.50%)	25/79 (31.62%)	
injection every 2 months	16/196 (8.16%)	10/117 (8.55%)	6/79 (7.59%)	
injection every 6 months	86/196 (43.88%)	49/117 (41.88%)	37/79 (46.84%)	
**Is there a pharmacological method to prevent HIV infection after exposure? [n/N (%)]**				
yes	114/196 (58.16%)	51/117 (43.59%)	63/79 (79.75%)	<0.001
no	17/196 (8.67%)	12/117 (10.26%)	5/79 (6.33%)	
I don’t know	65/196 (33.17%)	54/117 (46.15%)	11/79 (13.92%)	
**After a high-risk sexual encounter, would you like to take a medication that significantly reduces the risk of acquiring HIV? [n/N (%)]**				
yes	190/196 (96.94%)	112/117 (95.73%)	78/79 (98.73%)	0.404
no	6/196 (3.06%)	5/117 (4.27%)	1/79 (1.27%)	
**Have you ever heard of HIV PEP? [n/N (%)]**				
yes	97/196 (49.49%)	36/117 (30.77%)	61/79 (77.22%)	<0.001
no	99/196 (50.51%)	81/117 (69.23%)	18/79 (22.78%)	
**HIV PEP should be initiated within 72 h following unprotected sexual exposure [n/N (%)]**				
yes	111/196 (56.63%)	63/117 (53.85%)	48/79 (60.76%)	0.592
no	7/196 (3.57%)	4/117 (3.42%)	3/79 (3.80%)	
I don’t know	78/196 (39.80%)	50/117 (42.74%)	28/79 (35.44%)	
**Do you think that Doxy-PEP significantly reduces the risk of syphilis, chlamydia, or gonorrhea? [n/N (%)]**				
yes	102/196 (52.04%)	54/117 (46.15%)	48/79 (60.76%)	0.073
no	21/196 (10.71%)	12/117 (10.26%)	9/79 (11.39%)	
I don’t know	73/196 (37.24%)	51/117 (43.59%)	22/79 (27.85%)	
**Have you ever heard of Doxy-PEP? [n/N (%)]**				
yes	64/196 (32.65%)	21/117 (17.95%)	43/79 (54.43%)	<0.001
no	132/196 (67.35%)	96/117 (82.05%)	36/79 (45.57%)	
**Doxy-PEP significantly reduces the risk of infection with syphilis, chlamydia, or gonorrhea.** **[n/N (%)]**				
yes	166/196 (84.69%)	98/117 (83.76%)	68/79 (86.08%)	0.460
no	5/196 (2.55%)	2/117 (1.71%)	3/79 (3.80%)	
I don’t know	25/196 (12.76%)	17/117 (14.53%)	8/79 (10.13%)	
**Does doxy-PEP prevent from HIV? [n/N (%)]**				
yes	18/196 (9.18%)	12/117 (10.26%)	6/79 (7.59%)	0.178
no	137/196 (69.90%)	76/117 (64.96%)	61/79 (77.22%)	
I don’t know	41/196 (20.92%)	29/117 (24.76%)	12/79 (15.19%)	
**Following a high-risk sexual exposure, would you consider taking Doxy-PEP? [n/N (%)]**				
yes	166/196 (84.69%)	99/117 (84.62%)	67/79 (84.81%)	0.309
no	4/196 (2.04%)	1/117 (0.85%)	3/79 (3.80%)	
I don’t have an opinion	26/196 (13.27%)	17/117 (14.53%)	9/79 (11.39%)	
**Do you know where HIV PrEP, HIV PEP, or Doxy-PEP may be accessed? [n/N (%)]**				
yes	83/196 (42.35%)	39/117 (33.33%)	44/79 (55.70%)	0.002
no	113/196 (57.65%)	78/117 (66.67%)	35/79 (44.30%)	
**May HIV PrEP and Doxy-PEP be used simultaneously? [n/N (%)]**				
yes	75/196 (38.27%)	32/117 (27.35%)	43/79 (54.43%)	<0.001
no	6/196 (3.06%)	5/117 (4.27%)	1/79 (1.27%)	
I don’t know	115/196 (58.67%)	80/117 (68.38%)	35/79 (44.30%)	
**Do you think HIV PrEP should be reimbursed in Poland? [n/N (%)]**				
yes	123/196 (62.76%)	80/117 (68.38%)	43/79 (54.43%)	<0.001
no	16/196 (8.16%)	15/117 (12.82%)	1/79 (1.27%)	
I don’t know	57/196 (29.08%)	22/117 (18.80%)	35/79 (44.30%)	
**Rate your own knowledge of sexual health on a scale from 1 to 10.**				
median, [IQR]	7 [5–8]	6 [5–8]	8 [6–8]	0.002
**Do you think that proper sexual education can reduce STI incidence? [n/N (%)]**				
yes	188/196 (95.92%)	111/117 (94.87%)	77/79 (97.47%)	0.471
no	6/196 (3.06%)	4/117 (3.42%)	2/79 (2.53%)	
I don’t know	2/196 (1.02%)	2/117 (1.71%)	0/79	

**Abbreviations:** SMIs—Sexual minority individuals; k—thousand (inhabitants)**;** STIs—sexually transmitted infections; HIV PEP—HIV post-exposure prophylaxis; HIV PrEP—HIV pre-exposure prophylaxis; Doxy-PEP—doxycycline post-exposure prophylaxis.

**Table 2:** Univariable Poisson regression analyses of factors associated with awareness and knowledge of HIV PrEP, HIV PEP, and doxy-PEP among Polish adults by self-reported sexual orientation.

Outcome	Variable	PR	95% Cl	*p*
HIV testing	SMIs	2.27	(1.72–2.99)	<0.001
	Age	1.01	(0.99–1.02)	0.415
	Number of sexual partners	1.01	(1.01–1.02)	<0.001
	Male sex	1.83	(1.34–2.49)	<0.001
	Higher education	1.44	(1.05–1.97)	0.023
	Self-reported history of STI	2.16	(1.77–2.65)	<0.001
	Urban vs. rural residence	2.05	(1.13–3.72)	0.018
HIV PrEP awareness	SMIs	2.22	(1.72–2.86)	<0.001
	Age	1.00	(0.99–1.01)	0.663
	Number of sexual partners	1.01	(1.01–1.02)	<0.001
	Male sex	1.94	(1.44–2.61)	<0.001
	Higher education	1.22	(0.93–1.60)	0.146
	Previous HIV testing	2.51	(1.77–3.55)	<0.001
	Self-reported history of STI	1.85	(1.52–2.26)	<0.001
	Urban vs. rural residence	1.40	(0.91–2.16)	0.127
HIV PEP awareness	SMIs	2.51	(1.86–3.38)	<0.001
	Age	0.99	(0.98–1.01)	0.457
	Number of sexual partners	1.02	(1.01–1.02)	<0.001
	Male sex	1.86	(1.34–2.57)	<0.001
	Higher education	1.38	(1.01–1.91)	0.046
	Previous HIV testing	2.53	(1.72–3.72)	<0.001
	Self-reported history of STI	1.75	(1.35–2.27)	<0.001
	Urban vs. rural residence	1.47	(0.89–2.41)	0.129
Doxy-PEP awareness	SMIs	3.03	(1.96–4.69)	<0.001
	Age	1.01	(0.99–1.02)	0.454
	Number of sexual partners	1.02	(1.02–1.03)	<0.001
	Male sex	2.72	(1.64–4.50)	<0.001
	Higher education	1.71	(1.07–2.75)	0.026
	Previous HIV testing	3.30	(1.90–5.74)	<0.001
	Self-reported history of STI	2.27	(1.58–3.25)	<0.001
	Urban vs. rural residence	2.22	(0.97–5.08)	0.060
Self-reported history of STIs	SMIs	9.43	(3.90–22.83)	<0.001
	Age	1.01	(0.99–1.03)	0.577
	Number of sexual partners	1.03	(1.02–1.04)	<0.001
	Male sex	7.20	(2.69–19.29)	<0.001
	Higher education	1.34	(0.74–2.43)	0.340
	Previous HIV testing	28.21	(3.97–200.35)	<0.001
	Urban vs. rural residence	2.36	(0.79–7.08)	0.126
SMIs	Age	1.00	(0.98–1.01)	0.767
	Number of sexual partners	1.02	(1.02–1.03)	<0.001
	Male sex	4.64	(2.69–8.02)	<0.001
	Higher education	1.51	(1.02–2.23)	0.039
	Previous HIV testing	3.89	(2.26–7.00)	<0.001
	Self-reported history of STI	3.14	(2.31–4.28)	<0.001
	Urban vs. rural residence	2.78	(1.22–6.32)	0.015

**Abbreviations:** SMIs—sexual minority individuals, PR—prevalence ratio; CI—confidence interval.

**Table 3:** Multivariable Poisson regression analyses of factors associated with awareness and knowledge of HIV PrEP, HIV PEP, and doxy-PEP among Polish adults by self-reported sexual orientation.

Outcome	Variable	aPR	95% Cl	*p*
HIV testing	SMIs	1.44	(0.99–2.10)	0.058
	Age	1.00	(0.98–1.01)	0.692
	Number of sexual partners	1.00	(1.00–1.00)	0.881
	Male sex	1.11	(0.77–1.60)	0.568
	Higher education	1.34	(0.99–1.81)	0.060
	Self-reported history of STI	1.57	(1.25–1.97)	<0.001
	Urban vs. rural residence	1.55	(0.86–2.77)	0.143
HIV PrEP awareness	SMIs	1.46	(1.00–2.12)	0.050
	Age	0.99	(0.98–1.01)	0.422
	Number of sexual partners	1.00	(1.00–1.00)	0.342
	Male sex	1.29	(0.86–1.95)	0.201
	Higher education	0.89	(0.70–1.13)	0.350
	Previous HIV testing	1.97	(1.32–2.93)	<0.001
	Self-reported history of STI	1.02	(0.86–1.22)	0.792
	Urban vs. rural residence	0.90	(0.60–1.38)	0.636
HIV PEP awareness	SMIs	1.60	(1.04–2.45)	0.031
	Age	0.99	(0.98–1.01)	0.260
	Number of sexual partners	1.00	(1.00–1.01)	0.021
	Male sex	1.16	(0.73–1.84)	0.522
	Higher education	1.02	(0.74–1.41)	0.926
	Previous HIV testing	1.89	(1.20–2.96)	0.006
	Self-reported history of STI	0.92	(0.74–1.15)	0.482
	Urban vs. rural residence	1.08	(0.62–1.88)	0.791
Doxy-PEP awareness	SMIs	1.44	(0.82–2.50)	0.201
	Age	1.00	(0.98–1.02)	0.923
	Number of sexual partners	1.01	(1.00–1.02)	<0.001
	Male sex	1.33	(0.72–2.46)	0.362
	Higher education	1.07	(0.66–1.19)	0.794
	Previous HIV testing	2.12	(1.10–4.07)	0.025
	Self-reported history of STI	1.05	(0.71–1.54)	0.814
	Urban vs. rural residence	1.21	(0.51–2.86)	0.660
Self-reported history of STI	SMIs	3.68	(1.14–11.83)	0.029
	Age	1.02	(0.99–1.04)	0.116
	Number of sexual partners	1.01	(1.00–1.02)	0.027
	Male sex	1.60	(0.47–5.46)	0.456
	Higher education	0.69	(0.44–1.09)	0.110
	Previous HIV testing	13.68	(2.17–86.36)	0.005
	Urban vs. rural residence	0.62	(0.22–1.74)	0.359
SMIs	Age	0.981	(0.96–1.00)	0.039
	Number of sexual partners	1.00	(1.00–1.01)	0.321
	Male sex	3.995	(2.03–7.87)	<0.001
	Higher education	1.28	(0.90–1.81)	0.165
	Previous HIV testing	1.84	(1.01–3.33)	0.045
	Self-reported history of STI	1.48	(1.13–1.96)	0.005
	Urban vs. rural residence	1.56	(0.71–3.45)	0.267

**Abbreviations:** SMIs—sexual minority individuals; aPR—adjusted prevalence ratio; CI—confidence interval.

## Data Availability

The data sets used and/or analyzed during the current study can be made available by the corresponding author upon reasonable request. We are planning more analyses on this dataset; therefore, we can make it available on reasonable request.
